# Thermal Image Scanning for the Early Detection of Fever Induced by Highly Pathogenic Avian Influenza Virus Infection in Chickens and Ducks and Its Application in Farms

**DOI:** 10.3389/fvets.2021.616755

**Published:** 2021-05-25

**Authors:** Jin-Yong Noh, Kyu-Jik Kim, Sun-Hak Lee, Jun-Beom Kim, Deok-Hwan Kim, Sungsu Youk, Chang-Seon Song, Sang-Soep Nahm

**Affiliations:** ^1^Konkuk Ctc bio Animal Vaccine KCAV Co. Ltd, Seoul, South Korea; ^2^Department of Avian Disease, College of Veterinary Medicine, Konkuk University, Seoul, South Korea; ^3^Department of Anatomy, College of Veterinary Medicine, Konkuk University, Seoul, South Korea

**Keywords:** thermal imaging, highly pathogenic avian influenza, early detection, circadian rhythm, farm application

## Abstract

Highly pathogenic avian influenza (HPAI) is considered as one of the most devastating poultry diseases. It is imperative to immediately report any known outbreaks to the World Organization for Animal Health. Early detection of infected birds is of paramount importance to control virus spread, thus minimizing the associated economic loss. In this study, thermal imaging camera devices were used to detect change in the maximum surface temperature (MST) of chickens (*n* = 5) and ducks (*n* = 2) as an early indicator of experimental HPAI infection. The MST of both chickens and ducks increased at least 24 h before the manifestation of clinical signs of HPAI infection, depending on the severity of the infection. The basal MST was recorded for broiler chickens housed under small pen and normal farm conditions without intentional infection. A threshold cutoff of MST was established based on the circadian rhythm of normal MST. This study suggests that thermal imaging of chickens and ducks is a promising tool to screen any potential HPAI-infected flock in order to expedite HPAI diagnosis.

## Introduction

Highly pathogenic avian influenza (HPAI) is considered as one of the most devastating poultry diseases (1). HPAI virus infection in gallinaceous birds is associated with high mortality, often without any apparent clinical signs, imposing difficulties in controlling virus transmission prior to viral detection (2). Since 2003, seven HPAI outbreaks involving different clades of the H5 HPAI virus have occurred in South Korea; these outbreaks have been controlled by enhanced biosecurity measures and stamping-out policies (3, 4). For better implementation of preemptive measures, early disease detection and the subsequent reporting system should precede the virus spread (5). To date, the diagnosis of HPAI infection has mostly relied on passive surveillance upon suspicion and reporting by farmers or veterinarians. However, the absence of apparent clinical signs before sudden death poses difficulties in discriminating HPAI-induced death from daily mortality at the early stage of HPAI infection (5, 6).

A network of sensors refers to a variety of wireless micro-sensors attached to, or installed near, target objects to collect behavioral, biological, or modal information of individuals or groups, followed by its transmission to a comprehensive analytic system for risk assessment (7). Many studies have demonstrated the application of sensors in the poultry industry to improve flock management through the detection of abnormal night vocalization (8), jumping and landing force in laying hens (9), and floor distribution of broilers (10) under laboratory conditions. A thermal imaging device (TID) comprises a network of sensors that detect radiant heat from single or multiple target objects. As a non-invasive tool, its potential use has been investigated in the human and veterinary fields and confirmed for the detection of fever induced by pathogen infections or an increase in maximum surface temperature (MST) caused by heat stress or ventilation problems (11–14).

In the present study, we examined the potential use of TID to detect changes in MST associated with HPAI infection in chickens and ducks before the manifestation of clinical signs. For practical application, the MST of broiler chickens was monitored by TID over 4 weeks to establish a threshold cutoff of MST during their housing in simulated small pen and real broiler farm conditions with no intentional infection. In addition, real-time data collection and analyses were performed with a central analytic system to activate an alarm system when the monitored MST of chickens exceeded the threshold setup as per the experimental infection data.

## Materials and Methods

### Ethics Statement

All animal procedures performed in this study (permit number: KU18193) were reviewed, approved, and supervised by the Institutional Animal Care and Use Committee (IACUC) of Konkuk University.

### Viruses

All experiments with viable virus were conducted in biosafety level 3 and animal biosafety level (BL) 3 facilities at the Konkuk University. The HPAI H5N6 A/duck/Korea/ES2/2016(H5N6) strain was provided by the Animal and Plant Quarantine Agency, Korea, and propagated in 9–11-day-old specific pathogen-free (SPF) embryonated chicken eggs and stored at −70°C until further use.

### Thermal Imaging Analysis

Thermal video was recorded using an SM080TIP camera (Somo Energy & Technology Co., Ltd, Korea, Emissivity 0.98). Real-time changes in MST were monitored throughout the animal experiment. The MST was considered as one pixel representing the highest temperature among 80 × 60 pixels at each time point, regardless of the body part and number of animals. The MST of every 5 min was automatically transferred to a laptop via a network. The collected data of every 5 min were analyzed using the Argus viewer software program (Somo Energy & Technology Co., Ltd), and used for 3 h interval analyses (36 5-min time points for 3 h).

### Temperature Measurement Setup Under Normal Condition

First, 6-week-old SPF chickens and 5-week-old ducks were tested negative for influenza A virus using enzyme-linked immunosorbent assay (ELISA). To examine the compatibility of the TID in the experimental setup, five chickens and one duck, respectively, were monitored with an infrared camera in the BL2 facility at Konkuk University. The thermal images were taken into consideration for analysis based on the following criteria: (1) Which body area was the site for the recognition of the highest temperature? (2) Was there any difference in temperature monitoring between individual animals and groups of animals? (3) How does the distance between the TID and photographed animal affect the measured temperature?

### Temperature Measurement According to HPAI Infection

The five chickens used for the TID configuration were moved to negative pressure isolators in BL3. A thermal imaging camera was installed and fixed at the door of the isolator (0.3 m from the chickens) to minimize the effect of the distance between the camera and target object. MST was monitored at 5-min intervals. Basal MST was recorded from 24 h pre-challenge. The chickens were then intranasally challenged with 100 μL of H5N6 HPAI virus suspension (dose of 10^6.0^ EID_50_/bird). Changes in MST were compared with those reported before virus inoculation. Clinical signs and mortality were recorded daily, and swab samples were obtained at 40 h post-infection (hpi) to evaluate viral shedding. The lights were turned on and off every 12 h.

In addition, a separated duck experiment similar to the chicken experiment was conducted using two ducks. The ducks used for the TID configuration were intranasally challenged with 100 μL of two different doses of HPAI H5N6 virus (10^8.0^ EID_50_/bird, high dose; 10^4.0^ EID_50_/bird, low dose) to differentiate between the MST changes caused by higher and lower dose infection (15). MST was measured in the same way as that in chickens. Clinical signs and mortality were recorded daily, and swab samples were collected to quantify viral shedding at 2, 4, 7, 10, and 14 days post-infection (dpi). The temperature and relative humidity (20 ± 1°C and 50% humidity) of the animal facility were constantly maintained throughout the experiments.

### Virus Detection and Quantification

To investigate the replication of the virus inoculated in chickens and ducks, oropharyngeal and cloacal swab samples were collected as section Temperature Measurement According to HPAI Infection and re-suspended in 1 mL of sterile phosphate buffer solution. The suspension was centrifuged at 15,000 × g, and 200 μL of the supernatant was used for RNA extraction using MagNA Pure 96 DNA and Viral NA Small Volume Kit on a MagNA Pure 96 instrument (Roche Applied Sciences, Germany), according to the manufacturer's instructions. The amount of viral RNA was measured by real-time reverse-transcription quantitative polymerase chain reaction (RT-qPCR) and expressed as the cycle threshold (Ct) value (16).

### Maximum Surface Temperature Threshold Cut-Off Establishment in Actual Farm Setting

To establish a threshold cutoff for the MST in an actual farm setting, 30 1-day-old broiler chickens were raised in a 2 × 2 m pen for 4 weeks, thereby simulating a small-scale floor environment. For a larger scale environment, a broiler house with ~20,000 birds was monitored for the entire rearing period. Under simulated farm conditions, a thermal imaging camera was installed at a height of 2 m from the ground at a vertical shooting angle. In the broiler farm, a thermal imaging camera was installed at a height of 1.5 m from the ground near the farm door at a shooting angle of ~30° relative to the ground level (Supplementary Figure 1), unlike that in the case of the simulated farm. The temperature in a broiler house was changed from 33 to 23°C as per broiler growth and relative humidity was maintained at 50%. This monitoring was designed to set the threshold cutoff of the MST under farm conditions, considering environmental factors that affect MST, such as the circadian rhythm related to lighting.

### Statistical Analysis

For the challenge study, the 24 h temporal temperature data before challenge were averaged to set the basal MST and compared to the average of temperatures recorded at 3 h intervals following infection using one-way analysis of variance (ANOVA) with a Bonferroni *post-hoc* test. Time points with asterisks denote statistically different time points after infection. *P* < 0.05 were considered statistically significant.

## Results

### Temperature Measurement Under Normal Conditions

**Figure 1:**
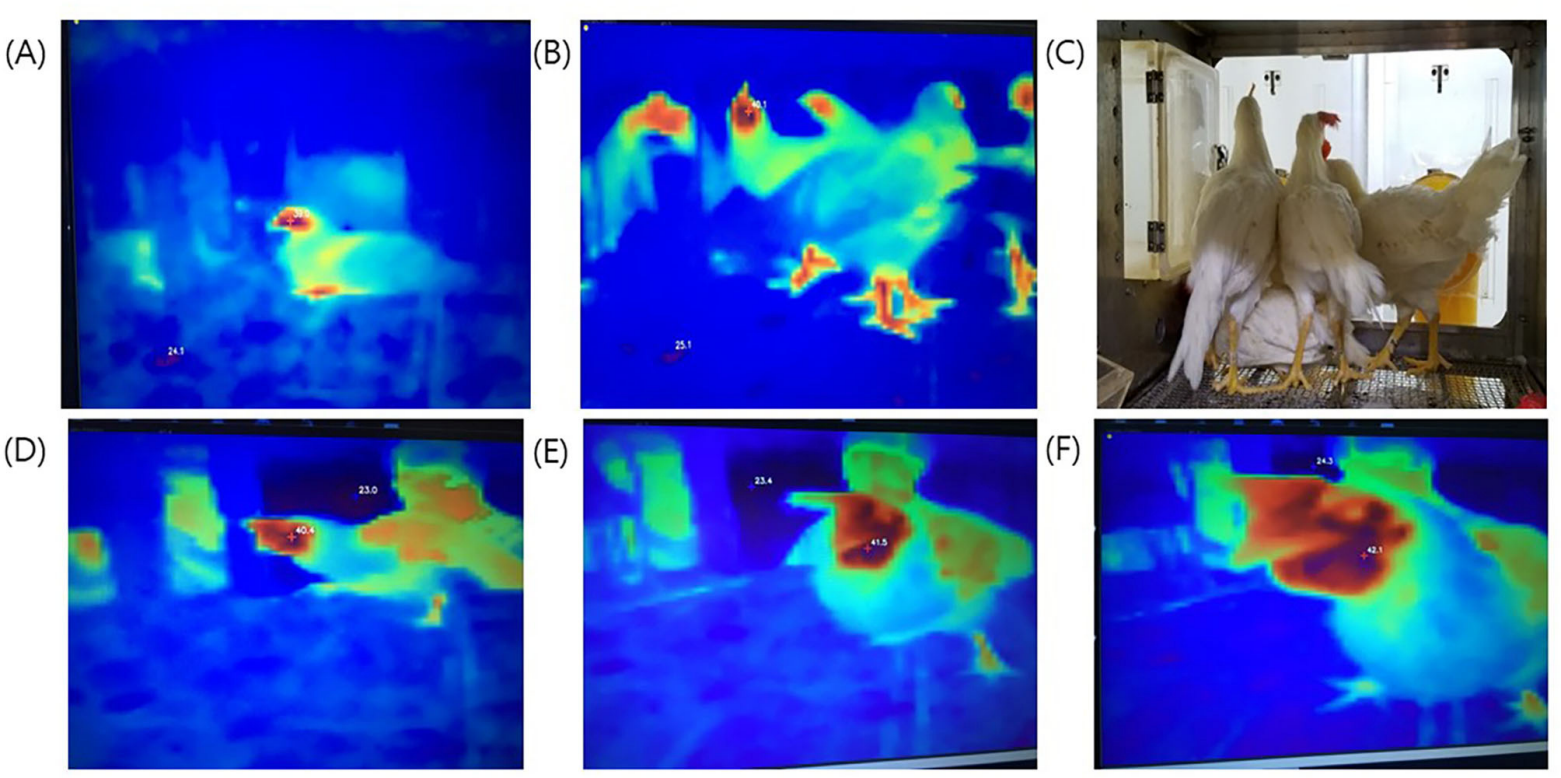
Thermal images taken from different experimental set-ups. Top photos **(A–C)**: Temperature measurement site of one chicken or several chickens compared to the actual photographs. **(A)** One chicken; **(B)** several chickens; **(C)** actual photo. Bottom photos **(D–F)**: Differences in temperature measurement relative to distance. The shorter the distance, the higher was the measured temperature. **(D)** long distance, 40.4°C; **(E)** intermediate distance, 41.5°C; **(F)** short distance 42.1°C.

We investigated the possibility of temperature detection using a thermal imaging camera by monitoring chickens and ducks before they were subjected to virus challenge. The MST was detected at the head and legs of the chickens, and the beak, wings, and legs of the ducks (Figure 1, Supplementary Figure 2). There was no interference effect in MST monitoring upon examination of a single bird or multiple birds. In addition, it was confirmed that TID could successfully identify the highest temperature among multiple birds, although the only one pixel representing highest temperature was sent to the laptop.

### Change in the Surface Temperature of Chickens After HPAI Virus Challenge

**Figure 2:**
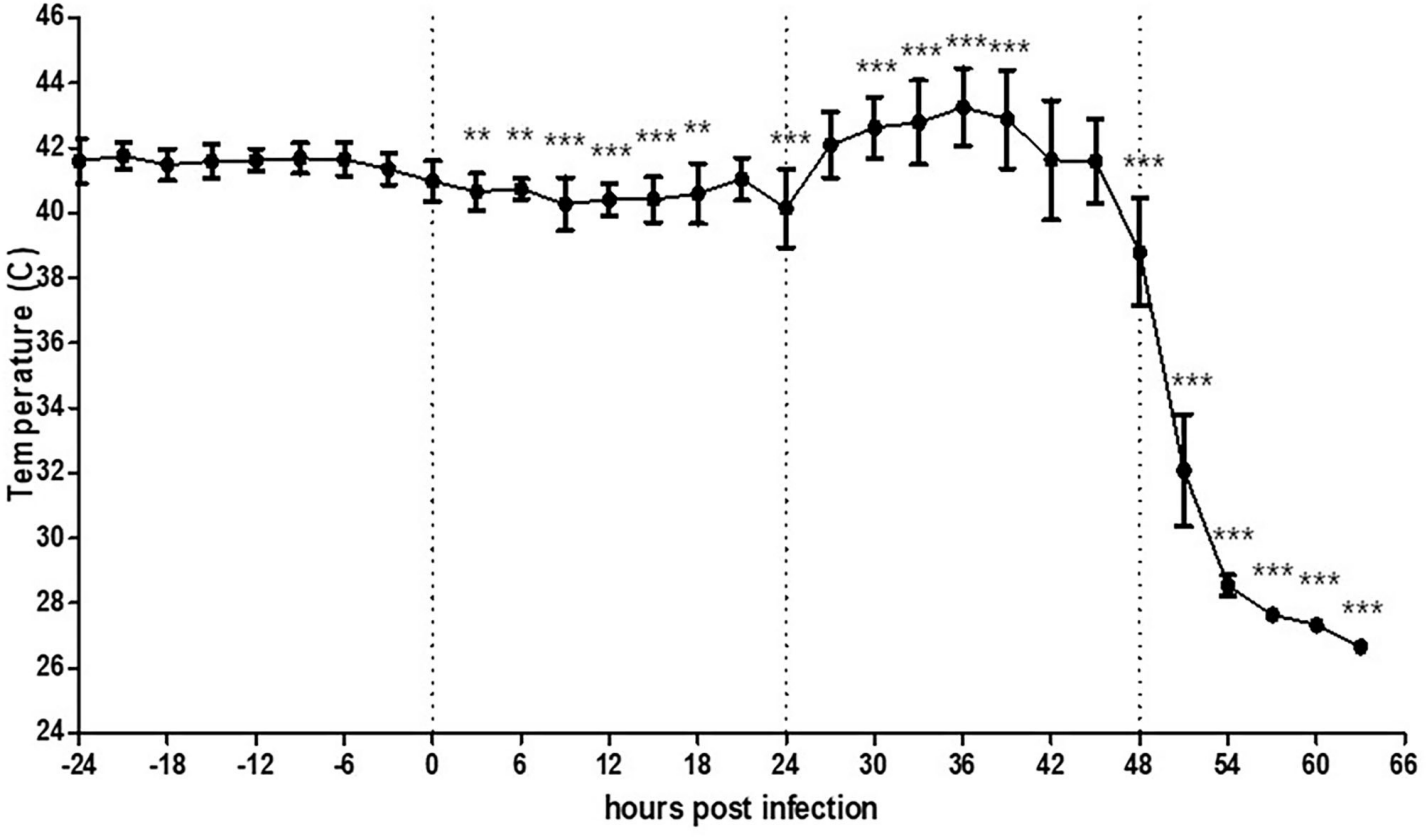
Average temperature per 3 h interval before and after HPAI virus challenge in chickens (*n* = 5). Chickens were further monitored several more hours after death without removal. The error bars represent standard deviations of 36 5-min time points. Statistical significance was determined using ANOVA followed by Bonferroni *post-hoc* test. ***P < 0.001; **P < 0.01.

To determine the effect of viral infection on the MST of chickens, we monitored their MST starting from 24 h before virus inoculation. The basal MST before viral infection varied from 40.5 to 42.4°C at each time point, and the mean maximum surface temperature (MMST) over time for 24 h was 41.6°C (Supplementary Figure 3). After virus inoculation, the MST decreased by as low as 1.3°C until 24 hpi The MST started increasing from 26 hpi and peaked to 42.9°C from 27 to 36 hpi (Figure 2), and suddenly decreased thereafter. This decrease in temperature was accompanied by lethargic behavior; the chickens finally died at 44 hpi. All chickens showed high viral shedding at 40 hpi from both the oropharynx (Ct = 25.7–30.5) and cloaca (Ct = 26.1–29.7).

### Change in the Surface Temperature of Ducks After HPAI Virus Challenge

The recorded basal MST of the two ducks at 5 weeks of age varied from 36.6 to 39.9°C (MMST = 38.2°C) and 38.7 to 41.8°C (MMST = 40.2°C) at each time point (Supplementary Figures 4, 5). This difference was not calibrated to body temperature because it had no effect on the monitoring of infection-related changes in MST. To demonstrate MST changes caused by either lethal or non-lethal infection of HPAI H5N6 virus, two different doses (10^8.0^ EID_50_/bird, high dose; 10^4.0^ EID_50_/bird, low dose) were used for the duck experiment.

**Figure 3:**
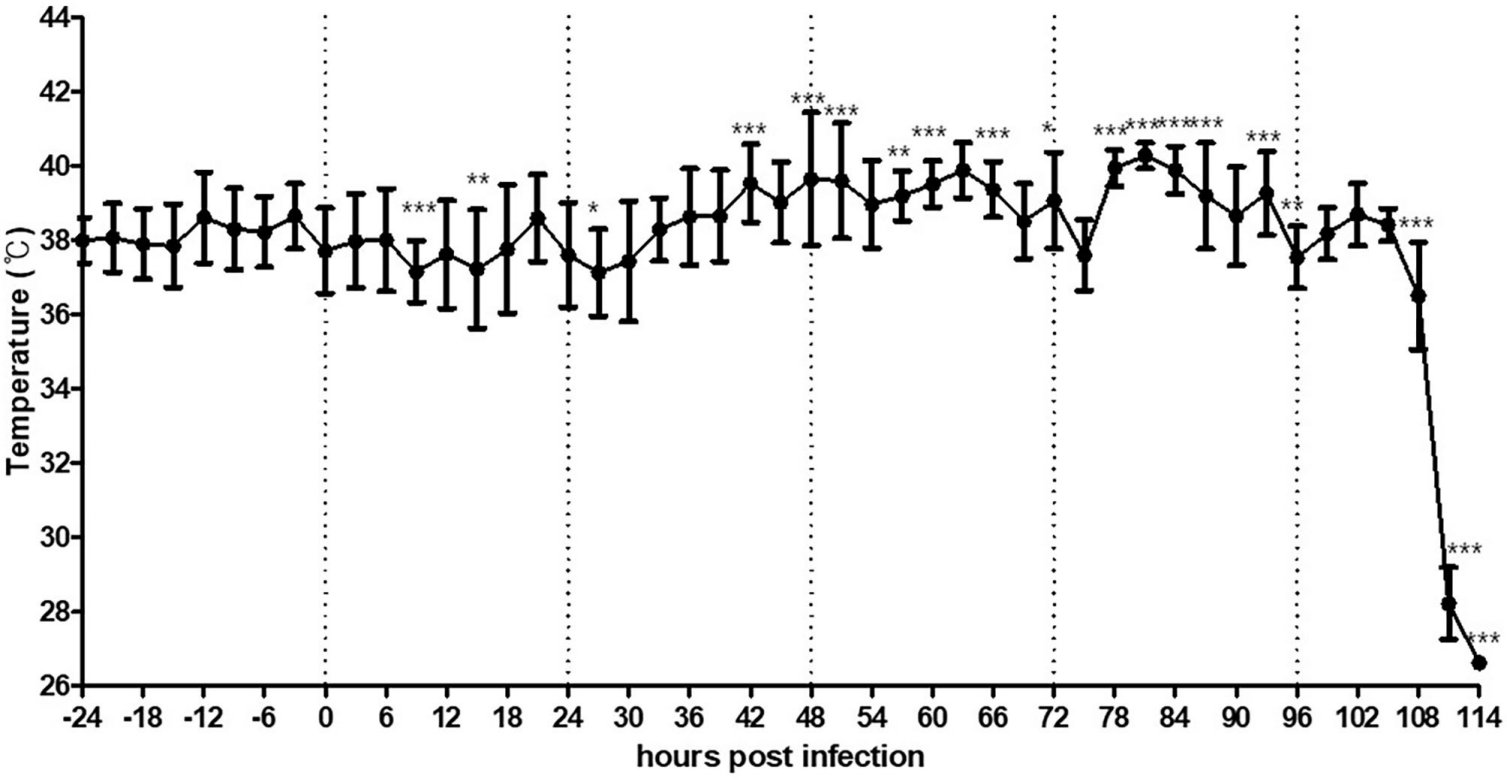
Average temperature per 3 h interval before and after HPAI virus challenge in a duck intranasally exposed to the virus at a dose of 10^8.0^ EID_50_/bird. Duck was further monitored several more hours after death without removal. The error bars represent standard deviations of 36 5-min time points. Statistical significance was determined using ANOVA followed by Bonferroni *post-hoc* test. ***P < 0.001; **P < 0.01; *P < 0.05.

The duck challenged with the high dose of the virus was found dead at 105 hpi. The dead duck showed a similar trend of increased MST as that observed in the chickens. After virus inoculation, the MST decreased by as low as 1.0°C until 27 hpi and started increasing from 40 hpi and peaked to 40.3°C from 80 to 86 hpi, which was 2.1°C higher than the MMST before viral challenge (Figure 3). The duck showed no obvious clinical signs before death, but viral shedding was detected in both oropharyngeal (Ct = 26.8 at 2 dpi and 30.8 at 4 dpi) and cloacal swabs (Ct = 31.2 at 2 dpi and undetermined at 4 dpi).

**Figure 4:**
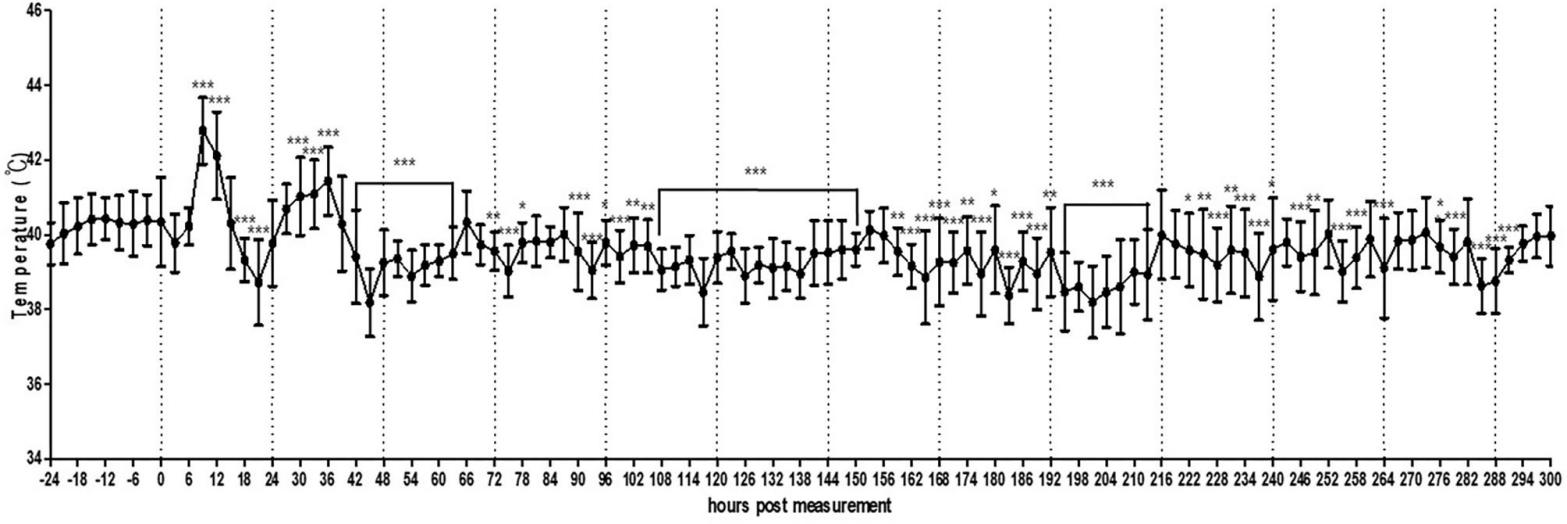
Average temperature per 3 h interval before and after HPAI virus challenge in a duck intranasally challenged with virus at 10^4.0^ EID_50_/bird dose. The error bars represent standard deviations of 36 5-min time points. Statistical significance was determined by ANOVA, followed by Bonferroni *post-hoc* test. ***P < 0.001; **P < 0.01; *P < 0.05.

The duck challenged with the low viral dose showed a bimodal temperature increase (42.8°C and 41.4°C, which was 2.6°C and 1.2°C higher than the MMST before viral challenge) and maintained its temperature below the basal MST after 39 hpi (Figure 4). Although the surviving duck showed no specific clinical signs, viral shedding was observed in oropharyngeal swabs (Ct = 31.6 at 2 dpi and undetermined at 4 dpi); this indicated that, viral infection was successfully performed.

### Chickens Housed Under Farm Conditions

**Figure 5:**
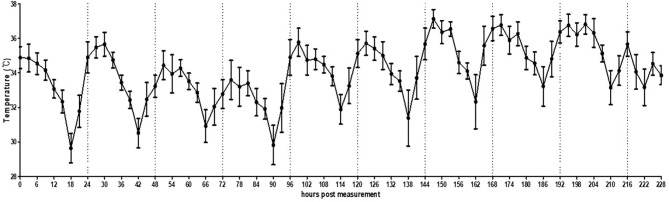
Average temperature per 3 h interval of broilers (*n* = 30) housed in a small pen size (2 × 2 m). The error bars represent standard deviations of 36 5-min time points.

In the small pen of the simulated floor farm conditions, the background temperature was too high to allow the recording of the MST of the chickens until the brooder was turned off. Therefore, the recording started once the heat lamp was turned off. It was confirmed that the MST of the chickens could be normally recorded even in a much wider environment than that of a small cage (Figure 5, Supplementary Figure 6). In addition, the circadian rhythm of MST was successfully detected using the thermal imaging camera by controlling the light at regular intervals. However, the recorded MST was lower than the actual MST and varied greatly from 28.0 to 38.0°C, thereby necessitating the calibration of image analysis.

**Figure 6:**
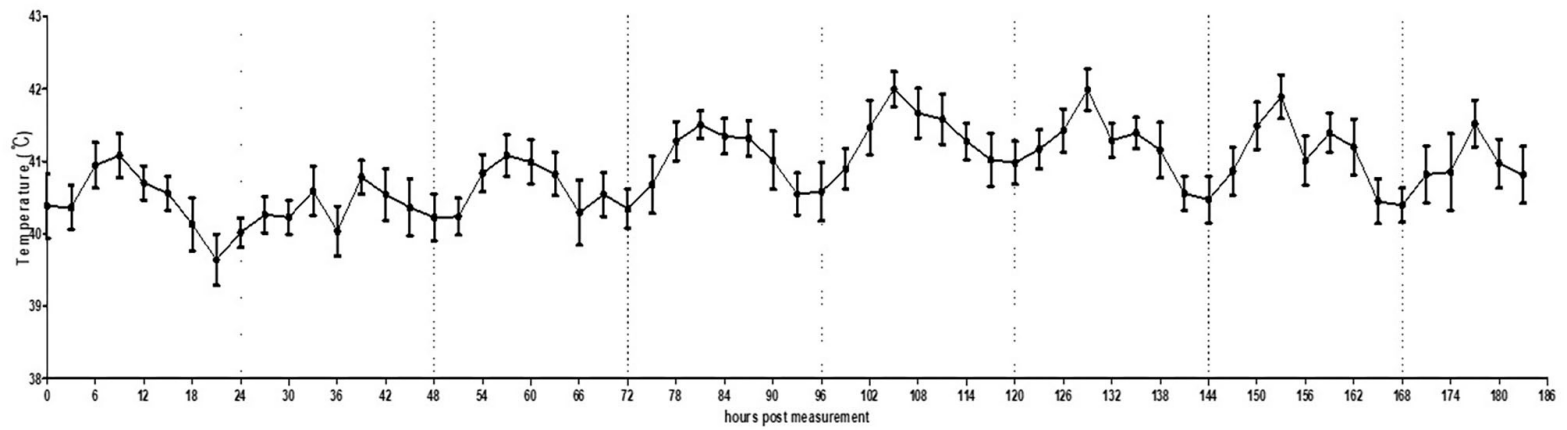
Average temperature per 3 h interval of broilers housed in an actual farm. The error bars represent standard deviations of 36 5-min time points. Thermography was performed for 7 days.

In the actual broiler farm condition, it was possible to normally shoot videos even when the brooder was turned on, as the brooder was installed on the ceiling and did not fall within the range of the thermal image. Consistent with the small pen simulation, the circadian rhythm of MST was observed under this actual broiler farm condition (Figure 6, Supplementary Figure 7). The temperature fluctuation was less than that in the small pen setting (39.0–42.0°C).

## Discussion

Thermal imaging cameras have been used for the screening of various infectious and non-infectious diseases in both human and veterinary fields, including the detection of severe acute respiratory syndrome (SARS) and coronavirus disease 2019 (COVID-19) (17, 18) in humans, bovine respiratory disease and bumblefoot in animals (19–21). In general, two different types of thermal data loggers have been devised for animal use, one of which is directly attached to the body and the other, placed at a certain distance to sense or image the surface temperature. A study showed that the attached sensors were able to precisely detect HPAI infection under experimental setup conditions (22). However, limited battery capacity and low coverage of data sampling are the main hurdles associated with the practical use of this technique. On the other hand, thermal imaging cameras can measure the temperature at a distance. The measured temperature may vary depending on the distance and humidity because the temperature recognition method involves the analysis of the infrared rays emitted from the object. However, the camera-type sensor is easy to maintain, and the calibration of the actual temperature based on the distance from which the image is obtained, as a default step, could minimize the variations in measurements. In addition, the use of thermal imaging cameras would be beneficial for monitoring the level of population in terms of flock-based surveillance for chickens and ducks.

In the present study, thermal imaging cameras could successfully record the temperature of an individual or community of birds in cage and broiler farm conditions and maintain a track of the patterns in MST fluctuations. It is well-documented that the MST of both chickens and ducks inoculated with a range of HPAI virus rises and is accompanied with active virus shedding early during the infection (22, 23). Hence, we aimed to detect the increased MST following HPAI infection. The MST was measured at the heads and legs of chickens and ducks, presumably owing to the presence of relatively fewer hair over these areas. In addition, the detected temperature varied with the distance between the thermal imaging camera and the object (Figure 1). Therefore, we set the normal temperature standard by shooting at a fixed measurement position to take the image in a uniform distance from 24 h before viral challenge or by continuous temperature monitoring for the farm experiment.

Throughout the recording period, different patterns in MST change were observed depending on the species, dose of virus inoculation, and severity of clinical signs. At high viral doses, both chickens and ducks succumbed to infection and showed a slight decrease in MST, which is statistically significant increased thereafter. This effect may be attributed to the inability of the body to function normally after initial infection, followed by fever related to the immune response. However, chickens showed lethargy a few hours before their death, whereas ducks did not show any clinical signs. As recording with thermal imaging cameras can detect statistically significant increase in MST at least 24 h before the death of an animal that the farmers usually recognize the infection, these results indicate that thermal imaging could serve as a useful strategy to rapidly predict HPAI-induced fever and minimize the response time to diagnosis or related preemptive measures. However, subclinical infection of ducks with low viral doses showed a different pattern than that of the lethal infection and might not be recognized until the subsequent virus spreads.

In the present study, we also recorded the pattern of change in basic temperature without intentional infection under farm conditions. Throughout the monitoring process, MST was not only influenced by disease conditions that induce fever but also by the circadian rhythm in a large number of birds under simulated and normal farm conditions. These circadian rhythm results will serve as the baseline under normal conditions, thereby allowing the tracking of temperature deviations induced by HPAI-induced fever. Thus, the alarming deviation in MST should be set in consideration of the daylight time and local temperature that affects the circadian rhythm of specific animals. In addition, the difference between the maximum and minimum temperatures in the simulated farm and actual farm conditions was very high, probably owing to the difference in the percentage of space used or shooting angle. The changes in the camera setting may have affected actual amplitude of minimum/maximum temperature within 24 h. Further vigorous experiments on the distance effect should be carried out to understand effects of shooting distance. Therefore, preliminary testing under normal conditions is essential for optimal calibration (for example, by comparing body temperature and TID; using more than one TID to monitor birds from different angles and making “stereo view” to apply triangulation).

Thermal imaging alone is inadequate for the diagnosis of any specific disease. However, the present study shows that thermal imaging can be used as an early monitoring tool for diseases such as HPAI. As a part of smart animal agriculture methods, thermal imaging could be applied across a wider range of applications for disease monitoring and detection of heat stress or ventilation problems for flock-based management.

## Data Availability Statement

The original contributions presented in the study are included in the article/Supplementary Material, further inquiries can be directed to the corresponding author/s.

## Ethics Statement

The animal study was reviewed and approved by Institutional Animal Care and Use Committee (IACUC) of Konkuk University. Written informed consent was obtained from the owners for the participation of their animals in this study.

## Author Contributions

J-YN, K-JK, and S-HL gathered the data for the study and conducted the data analysis. J-YN prepared the manuscript. SY contributed to the interpretation of the results and manuscript preparation. J-BK contributed to the statistical analysis. D-HK coordinated the sample submission and testing. C-SS and S-SN conceived and supervised the study. S-SN reviewed, edited, and approved the manuscript. All authors contributed to the article and approved the submitted version.

## Conflict of Interest

J-YN, K-JK, J-BK, and C-SS were employed by KCAV Co. Ltd. The remaining authors declare that the research was conducted in the absence of any commercial or financial relationships that could be construed as a potential conflict of interest.
