# Supplementary figures and images for: Thermal Image Scanning for the Early Detection of Fever Induced by Highly Pathogenic Avian Influenza Virus Infection in Chickens and Ducks and Its Application in Farms
Source: Front Vet Sci. 2021 May 25;8:616755. doi: 10.3389/fvets.2021.616755 (PMC8185153; doi:10.3389/fvets.2021.616755)

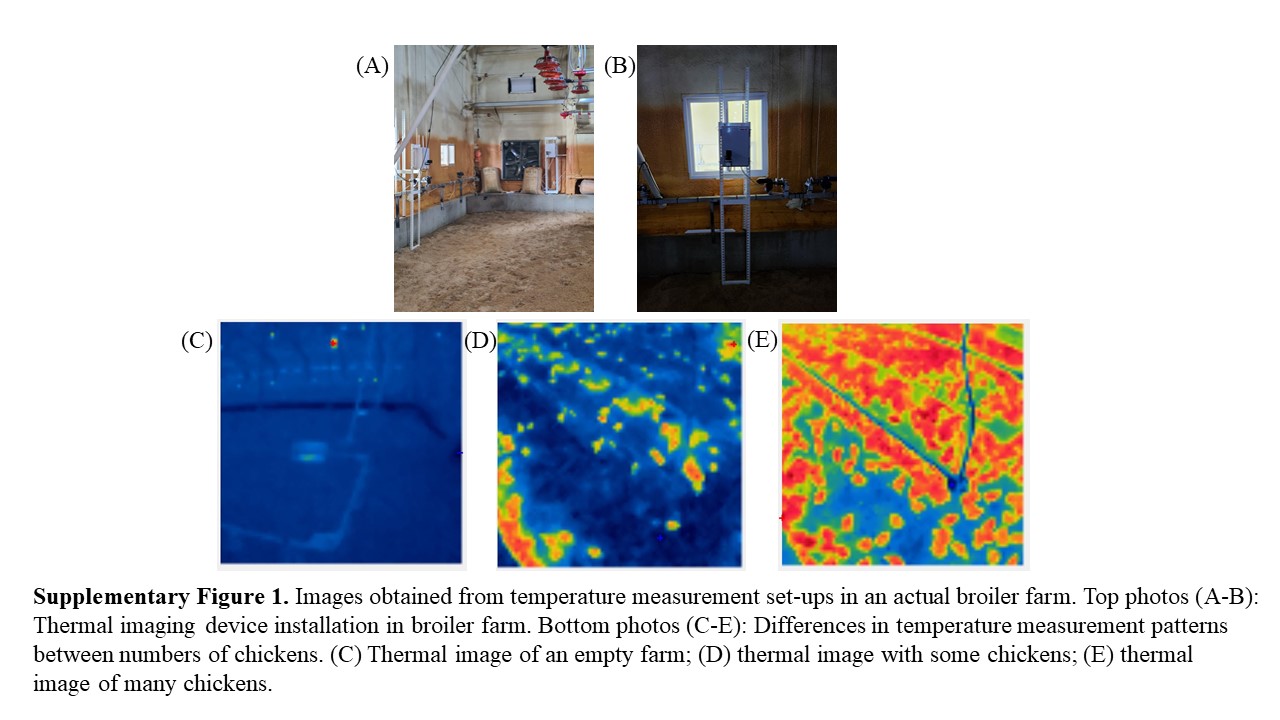

Supplement: Supplementary file 1 [file Image_1.JPEG]

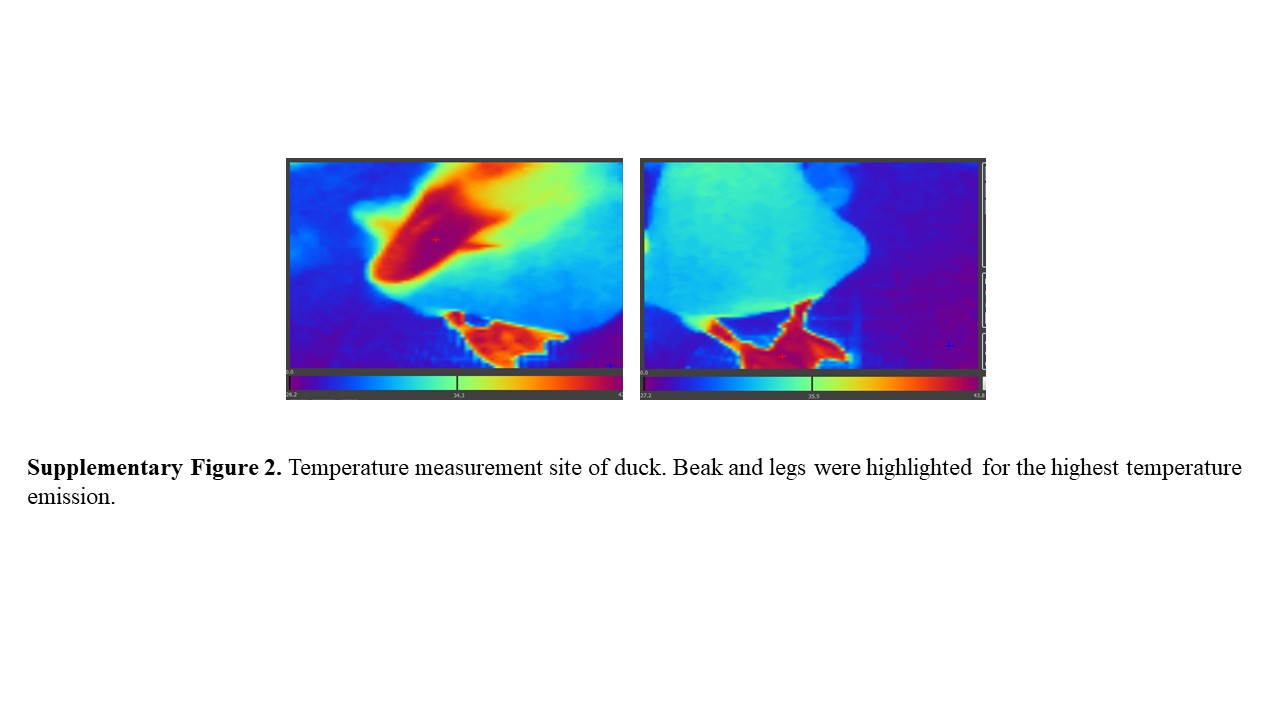

Supplement: Supplementary file 2 [file Image_2.JPEG]

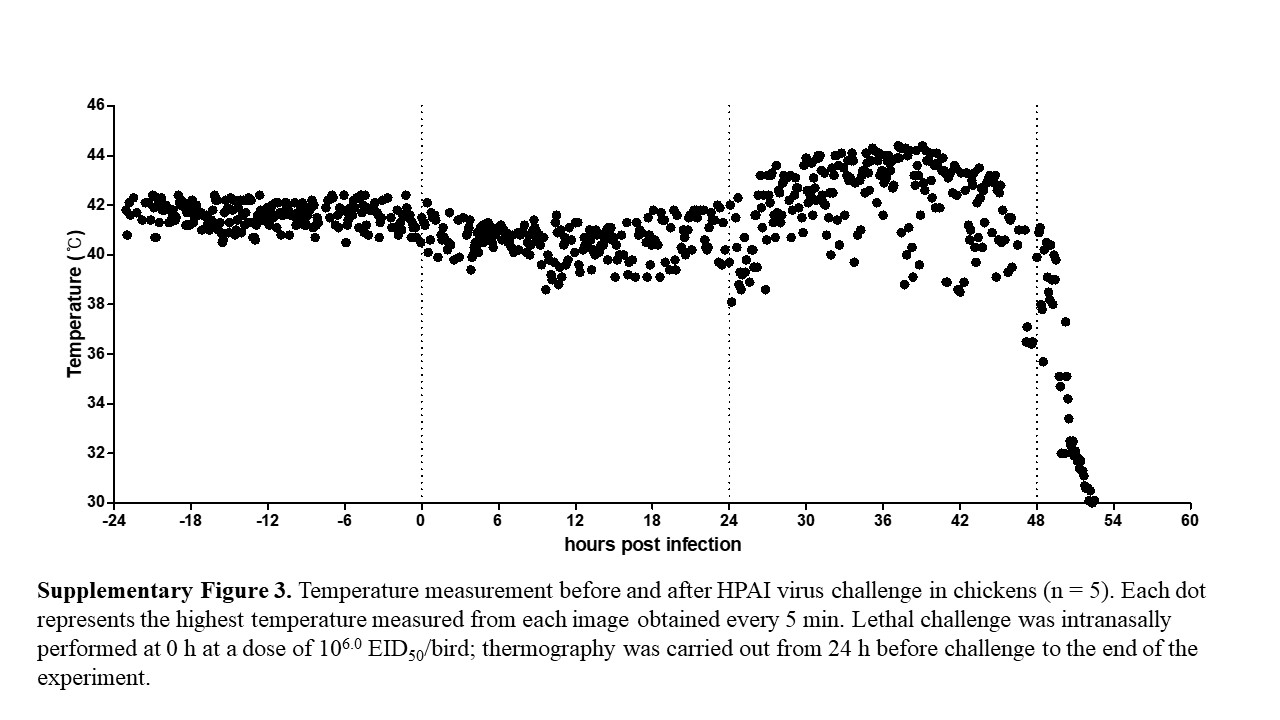

Supplement: Supplementary file 3 [file Image_3.JPEG]

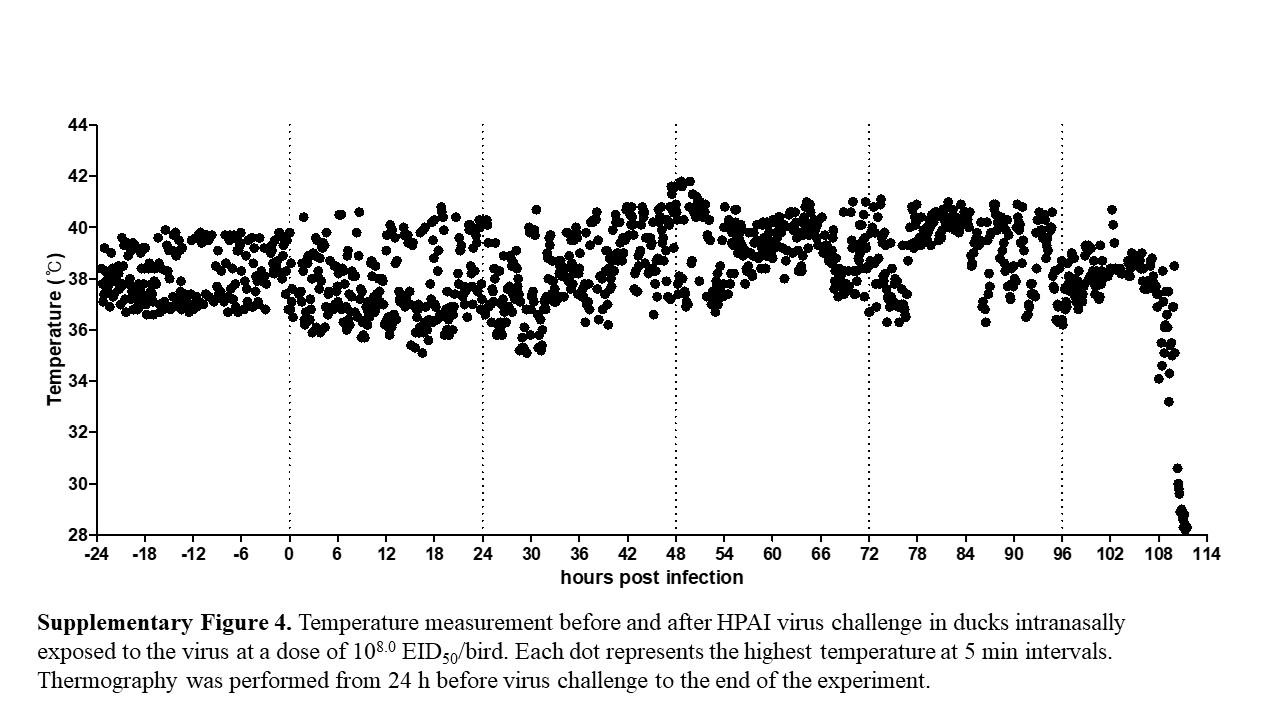

Supplement: Supplementary file 4 [file Image_4.JPEG]

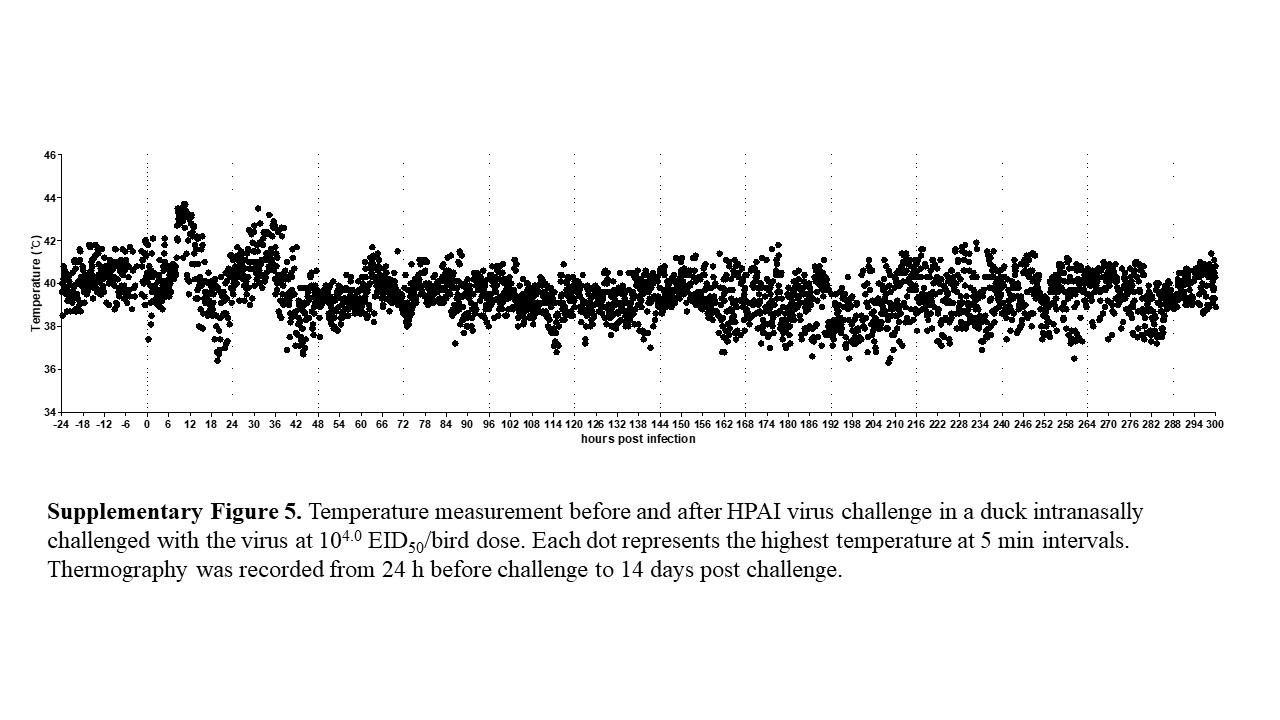

Supplement: Supplementary file 5 [file Image_5.JPEG]

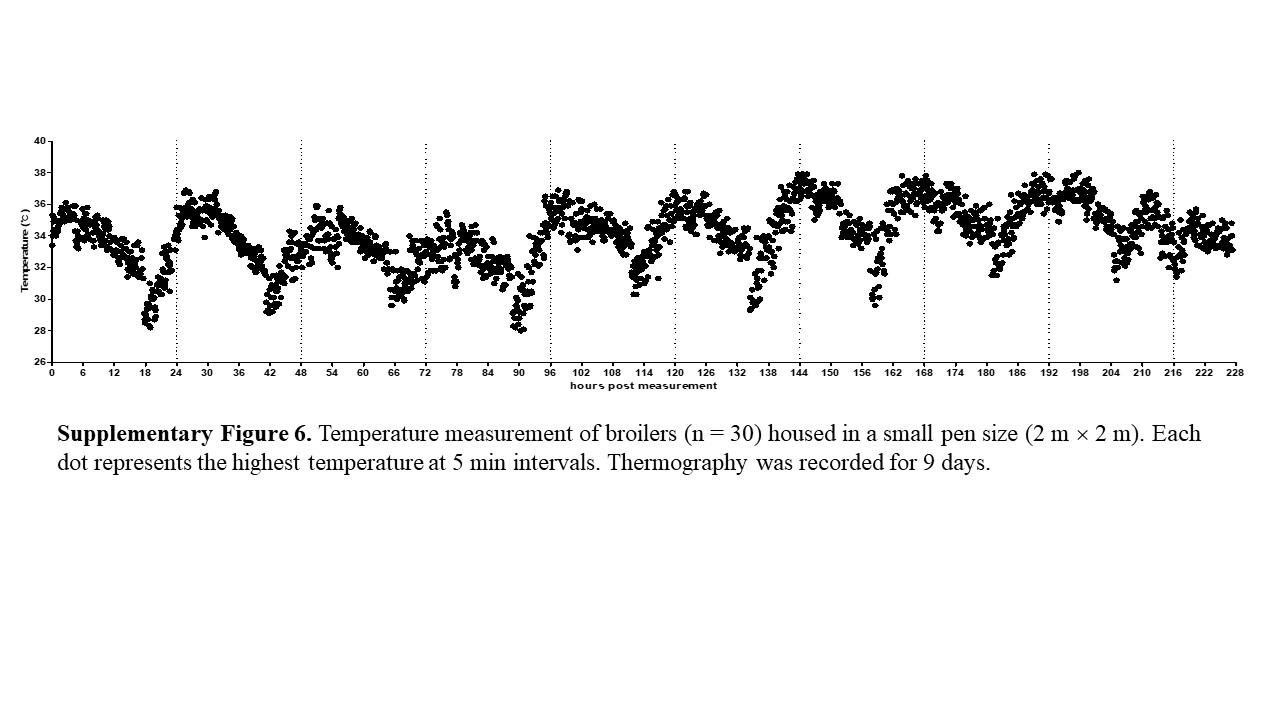

Supplement: Supplementary file 6 [file Image_6.JPEG]

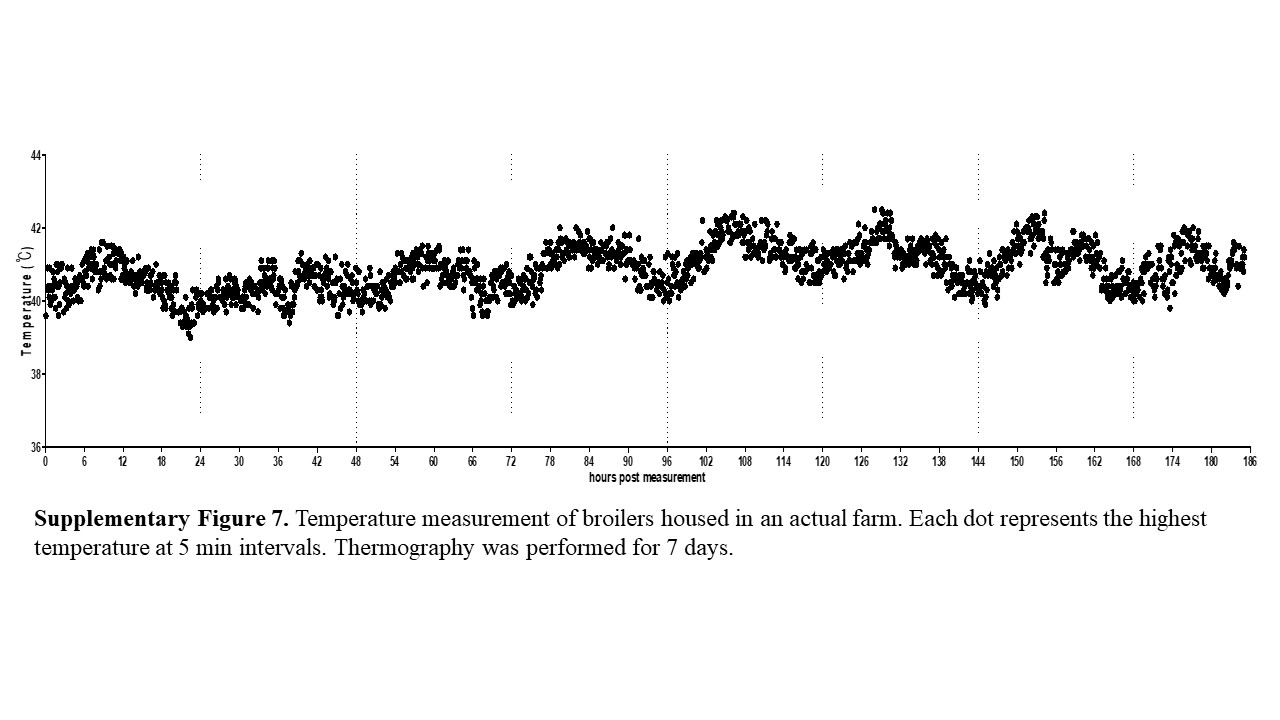

Supplement: Supplementary file 7 [file Image_7.JPEG]
